# Management of post-traumatic stress disorder: A protocol for a multiple treatment comparison meta-analysis of randomized controlled trials

**DOI:** 10.1097/MD.0000000000017064

**Published:** 2019-09-27

**Authors:** Yasir Rehman, Behnam Sadeghirad, Gordon H. Guyatt, Margaret C. McKinnon, Randi E. McCabe, Ruth A. Lanius, Donald J. Richardson, Rachel Couban, Helena Sousa-Dias, Jason W. Busse

**Affiliations:** a Department of Health Research Methods, Evidence, and Impact (HEI); b The Michael G. DeGroote Institute of Pain Research and Care, McMaster University; c Canadian Academy of Osteopathy (CAO); d Department of Psychiatry and Behavioral Neurosciences, McMaster University; e Mood Disorders Program, St. Joseph's Healthcare Hamilton, Hamilton; f Homewood Research Institute, Guelph; g Anxiety Treatment & Research Clinic, St. Joseph's Healthcare Hamilton, Hamilton; h Imaging Division, Lawson Health Research Institute; i Department of Psychiatry and Neurosciences Western University; j MacDonald/Franklin OSI Research Centre, Western University; k Lawson Health Research Institute; l Parkwood Hospital Operational Stress Injury Clinic, St. Joseph's Health Care London; m Department of Psychiatry and Neurosciences Western University, London; n Oakville; o Department of Anesthesia; p The Michael G. DeGroote Centre for Medicinal Cannabis Research, McMaster University, Hamilton, ON, Canada.

**Keywords:** CAPS, disability, functions, network meta-analysis, PTSD, quality of life, return to work

## Abstract

**Background:**

Most systematic reviews have explored the efficacy of treatments on symptoms associated with post-traumatic stress disorder (PTSD), which is a chronic and often disabling condition. Previous network meta-analysis (NMA) had limitations such as focusing on pharmacological or psychotherapies. Our review is aims to explore the relative effectiveness of both pharmacological and psychotherapies and we will establish the differential efficacy of interventions for PTSD in consideration of both symptom reduction and functional recovery.

**Methods:**

We will conduct a network meta-analysis of randomized controlled trials evaluating treatment interventions for PTSD. We will systematically search Medline, PILOT, Embase, CINHAL, AMED, Psychinfo, Health Star, DARE and CENTRAL to identify trials that: (1) enroll adult patients with PTSD, and (2) randomize them to alternative interventions or an intervention and a placebo/sham arm. Independent reviewers will screen trials for eligibility, assess risk of bias using a modified Cochrane instrument, and extract data. Our outcomes of interest include PTSD symptom reduction, quality of life, functional recovery, social and occupational impairment, return to work and all-cause drop outs.

**Results:**

We will conduct frequentist random-effects network meta-analysis to assess relative effects of competing interventions. We will use a priori hypotheses to explore heterogeneity between studies, and assess the certainty of evidence using the GRADE approach.

**Conclusion:**

This network meta-analysis will determine the comparative effectiveness of therapeutic options for PTSD on both symptom reduction and functional recovery. Our results will be helpful to clinicians and patients with PTSD, by providing a high-quality evidence synthesis to guide shared-care decision making.

## 1 Introduction

Post-traumatic stress disorder (PTSD) results from experiencing or witnessing an emotionally traumatic event that is perceived to present a threat to the life or physical integrity of one's self or others. PTSD is defined by 8 criteria **(**Table 1),^[1,2]^ including exposure to a traumatic event and resulting symptoms from each of 4 clusters:

(1) intrusion,
(2) avoidance,
(3) negative alterations in cognitions and mood, and
(4) alterations in arousal and reactivity.

**Table 1:**
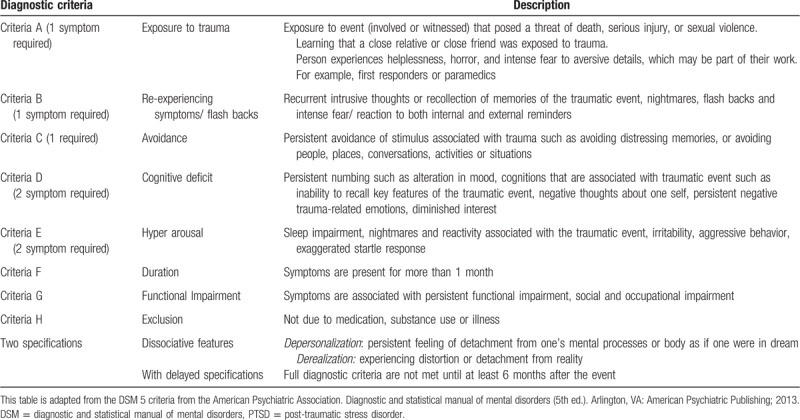
DSM 5-criteria for PTSD with description.

The sixth criterion requires that symptoms have been present for >1 month; the seventh assesses functioning (ie, symptoms interfere with the ability to go about normal daily tasks); and the eighth criterion is that symptoms are not attributable to another medical condition.^[3]^ Whereas some symptoms associated with PTSD (eg, hypervigilance) function to maintain an ongoing sense that the inciting event could happen again, which in turn, maintains fear and avoidance,^[4]^ others are associated with persistent feelings of shame and guilt (eg, negative-trauma related emotions). The diagnostic and statistical manual of mental disorders (DSM-5)^[3]^ also includes a dissociative subtype of PTSD, in recognition that 15% to 30% of patients with PTSD presents with symptoms of depersonalization and derealization.^[5–15]^

The lifetime prevalence of PTSD among the general public is 8% to 9%,^[16–18]^ and between a quarter and a third of workers exposed to traumatic events develop symptoms of PTSD.^[19,20]^ Patients suffering with PTSD experience social impairment, high absenteeism, unemployment^[21–27]^ and work-related disability, particularly when completing tasks requiring high concentration and cognitive demands.^[24–25]^ According to a 2016 review, 2.5 million of the general population and 70,000 first responders in Canada would suffer with PTSD in at some point in their lifetime.^[28]^ In a retrospective study of 44 PTSD claims awarded by the Workers Compensation Board of British Columbia, Canada, only 43% of disabled workers returned to their previous job 4 years after their trauma, 23% returned to alternate employment, and 34% did not returned to any type of work.^[29]^ Similarly, a prospective study of 94 PTSD patients in Ontario, Canada, found 57% had not returned to work in any capacity 9 months after their motor vehicle accident; among the 43% who had, a little more than half (57%) required job modification.^[30]^

There is uncertainty regarding what treatments are most effective for facilitating return-to-work among traumatized workers. Existing systematic reviews focus on the effect of selected therapies instead of exploring the relative effectiveness of competing therapies, and on amelioration of PTSD symptoms versus functional recovery.^[31–37]^ A previous network meta-analysis (NMA) of pharmacological treatments for PTSD^[38]^ has a number of limitations, including:

(1) an outdated search (February 2016),
(2) consideration of pharmacologic therapies only,
(3) narrow focus on exploring treatment effects for only symptom reduction and all-cause drop-outs, and
(4) use of the surface under the cumulative ranking curve (SUCRA) approach to rank interventions – an approach that does not consider the quality of evidence, or the estimates of precision around treatment effects.

Another NMA^[39]^ was similarly limited by a narrow focus only on psychotherapeutic approaches, an outdated search (January 2011), time-restricted search strategies (1980–2010), inclusion of trials with patients who had subclinical or sub-threshold PTSD and treatment effects limited to only symptom reduction.

As existing reviews of treatment for PTSD have largely focussed on individual treatment's effect on symptom reduction and none have compared all treatments against each other. Due to which there exist considerable uncertainty regarding what treatments are effective for promoting symptom control and functional recovery. Our proposed NMA will evaluate all treatments for PTSD, provide relative effectiveness of treatments, and evaluate the quality of the evidence in a thorough and consistent manner using the grading of recommendations assessment, development, and evaluation (GRADE) approach.

## 2 Methods

We registered our protocol on PROSPERO (CRD42018072682). Our review will conform to the PRISMA Extension Statement for Reporting of Systematic Reviews Incorporating Network Meta-analyses of Health Care Interventions.^[40]^

### 2.1 Search strategy

We identified all relevant randomized trials, in any language, through a systematic search of published international literature on traumatic stress, CINAHL, EMBASE, MEDLINE, AMED allied and complementary medicine, HealthSTAR, database of abstracts of reviews of effects, PsychINFO, and the Cochrane Central Registry of Controlled Trials (Appendix#1). An experienced medical librarian refined our search strategy for each database. Reviewers scanned the bibliographies of all eligible trials and other relevant publications for additional trials. We did not have any language restriction.

### 2.2 Eligibility criteria and study selection

Trials were eligible if they enrolled adult patients diagnosed with primary or secondary PTSD according to operationalized criteria such as DSM criteria, international classification of diseases criteria, or clinician diagnosed and randomized them to any pharmacologic (monotherapy or add-on therapy) or nonpharmacologic treatment strategy compared to an alternative treatment, their combinations, placebo or control. Eligible pharmacologic interventions included but were not restricted to antidepressants such as selective serotonin inhibitor, nonselective serotonin inhibitors, anxiyoltics, antipsychotics, and antiepileptics or mood stabilizers. Eligible nonpharmacologic interventions included but were not restricted to: cognitive behavioral therapy, exposure therapy, yoga, supportive therapy, and eye movement desensitization and reprocessing.

We will exclude trials with less than 10 participants per arm, pilot or feasibility or preliminary studies, and cross-over trials. We will also exclude trials in which the population does not constitute 100% PTSD patients including sub-threshold or subclinical or partial PTSD. We will also exclude studies that enrolled patients suffering with acute stress disorders. Conference abstracts and rarely used interventions or interventions that are discontinued due to serious adverse effects (such as Nefazadone, Brofaromine) rarely used for interventions in North America will be excluded.

Using standardized forms, reviewers will work independently in duplicate to screen title and abstracts, and the full text of all potentially eligible studies. Before starting literature screening, all reviewers will complete a pilot exercise to ensure understanding of the process and improve reliability. Any discrepancies will be resolved through consensus between reviewers or with the help of an adjudicator.

### 2.3 Data abstraction

To help ensure the reliability of independent data extraction, we will begin by piloting our data extraction form and then we will conduct calibration exercises between reviewers. Data abstraction will be guided by a detailed instruction manual generated from our piloting and calibration exercises. Teams of reviewers will extract data independently and in duplicate from eligible trials and resolve discrepancies through discussion. Data abstracted will include study characteristics (the first author, publication year, funding source); patient and trial characteristics such as sample size, participant demographics (age, gender, litigation and disability status of the participants, associated co-morbid psychological and substance status, traumatic brain injury and first responder status such as veterans, police officers and fire fighters); characteristics of interventions and comparators (dose of pharmacological treatments, frequency for nonpharmacologic treatments, use of other intervention such as medications or psychotherapies during the trial).

We will extract data on the on return to work (percent patients on partial or complete sick leave, percent patients with partial or complete functional recovery). We will also extract data on the PTSD symptoms such as reported with clinician-administered PTSD scale (CAPS),^[41–43]^ PTSD checklist^[44]^ or any other validated scales, quality of life such as short-form 36 with Physical (PCS) and mental (MCS) component summaries,^[45]^ or any other validated quality of life scale such as the World Health Organization Quality of Life Questionnaire,^[46]^ The Quality of Life Enjoyment and Satisfaction Questionnaire^[47]^; functional impairment with Sheehan's disability scale,^[48]^ and social and occupational impairment with any validated scale such as social adjustment scale.

If an individual trial reports more than 1 outcome in a common domain (eg, return to work), we will select only 1 outcome per domain based on the following criteria:

(1) most commonly used outcome measure across eligible trials;
(2) most validated outcome; or
(3) most precise estimate of treatment effect.

After consulting with our expert panel members, the CAPS will be preferred over any other scale for assessing PTSD symptoms. In the case of multiple follow-ups, we will collect data on the longest follow-up.

### 2.4 Risk of bias assessment

Reviewers will assess risk of bias among the eligible studies using the Cochrane risk of bias instrument that has modified response options of “definitely or probably yes” – considered as low risk of bias – or “definitely or probably no” – considered as high risk of bias.^[49]^ We will evaluate the following risk of bias issues: random sequence generation, allocation concealment, blinding of study participants, personnel, outcome assessors, and data analysts; selective reporting, and incomplete outcome data (>20% missing participant's data).^[50]^ We will assess risk of bias for each outcome separately on a component-by-component basis.^[51]^

### 2.5 Data synthesis

We will do drug adjudication to collapse similar interventions or drugs belonging to the same class into 1 treatment node. For dichotomous outcomes (eg, percent patients on partial or complete sick leave, percent patient with partial or complete functional recovery), we will calculate relative risk and absolute risk (using baseline risk estimates from the control arm of eligible studies), and the associated 95% confidence intervals (CIs) for outcomes. For studies reporting continuous outcomes (eg, PTSD symptom severity, quality of life such as SF-36 (PCS and MCS), disability, social, and occupational impairment when the same instrument is used, we will calculate the weighted mean difference, and the associated 95% CIs. For trials that use different instruments for the same underlying construct such as functional recovery, we will convert all outcomes to a common instrument based on the recommendations by Thorlund et al.^[52]^

We will contact study authors in case data is not completely reported or will use methods suggested by Cochrane handbook^[53]^ and Hozo et al^[54]^ to impute missing standard deviations when *P*-values, *t*-values, CIs, range, or standard errors (SEs) are reported in articles.

### 2.6 Methods for direct comparisons

We will perform standard pairwise meta-analyses using the DerSimonian–Laird random-effects model for all outcomes with at least 2 studies.^[55]^ We will determine statistical heterogeneity using the *Q* statistic and *I*^2^. For each direct comparison, we will report study and participant characteristics, risk of bias findings, and pooled estimates for outcomes of interest.

### 2.7 Methods for multiple treatment comparisons (NMA)

To assess the comparative effectiveness of competing treatments, we will perform random-effects NMA using the frequentist approach.^[56–59]^

Although the assumptions for NMA are similar to conventional meta-analysis, key extra assumptions are transitivity (there are no effect modifiers influencing the indirect comparisons) and coherence, (direct and indirect effect estimates are similar).^[60]^ We will identify incoherence comparing direct evidence (ie, estimates from pairwise comparisons) with indirect evidence (ie, estimates form network meta-analysis) using the node splitting method. In this approach, the incoherence will be assessed locally within each closed loop of the network separately as the difference between direct and indirect estimates for a specific comparison in the loop.^[56–57]^ We will use a Wald test to test any statistical difference between the direct and the indirect estimates.^[57]^

We will report our findings with probability statements of intervention effects. Probability rankings allow us to report a chance percentage of which interventions rank higher; however, simplifying the results of a network down to probabilities can lead to misinterpretations, specifically, when particular comparisons (ie, nodes) are not well-connected and/or when certainty in evidence varies between comparisons. Following display of the rank probabilities using rankogram, we will use the SUCRA to aid in the interpretation of relative effect of the interventions; an intervention with a SUCRA value of 100 is certain to be the best, whereas an intervention with 0 is certain to be the worst.^[52]^ We will use STATA (StataCorp, Release 15.1, College Station, TX) for statistical analyses.

### 2.8 Certainty (quality) of evidence

We will use the GRADE approach to rate the certainty in evidence of direct, indirect, and network estimates^[61–64]^ on an outcome-by-outcome basis that classifies evidence as “high,” “moderate,” “low,” or “very low.” The starting point for certainty in estimates from randomized trials is high, but maybe rated down based on limitations in risk of bias, imprecision, inconsistency, and indirectness, and publication bias.^[63]^

We will follow detailed guidance that the GRADE working group has provided for rating the quality of treatment effect estimates from network meta-analysis.^[62]^ In brief, the rating consists of 4 steps: first, we will present direct and indirect treatment estimates for each comparison of the evidence network. The direct estimate of effect is provided by a head-to-head comparison (trials of A vs B), and the indirect estimate is provided by 2 or more head-to-head comparisons that share a common comparator (for example, we infer the effects of A vs B from trials of A vs C and trials of B vs C). Second, we will rate the quality of each direct and indirect effect estimate; third, we will present the NMA estimate for each comparison of the evidence network and finally, we will rate the quality of each NMA effect estimate.

We will base hierarchy of the treatment options in the NMA according to 3 categories:

(1) those that are clearly superior;
(2) those with intermediate effectiveness, and
(3) those that are inferior.

Treatments no better than placebo will be categorized in the lowest tier; treatments better than placebo will be categorized in the intermediate tier; whereas those superior to at least 1 tier 1 treatment will be judged superior. Furthermore, treatments will be categorized according to the quality of evidence such as high and moderate versus low or very low. Interventions with high quality or moderate evidence will be ranked as either “among the most effective,” “inferior to the most effective/superior to the least effective,” or “among the least effective.” Interventions supported by low or very low quality evidence will be ranked into the same 3 categories but prefaced with “may be” to acknowledge the reduced confidence in supporting evidence (eg, “may be among the most effective”) and will be presented separately from those supported by moderate or high quality evidence.

We will assess small study effects in direct comparisons using the Harbord test for dichotomous outcomes and the Egger test for continuous outcome when at least 10 studies are available.^[62,64]^

### 2.9 Subgroup and sensitivity analyses

We will test 7 a priori hypotheses to explain variability between studies:

(1) patients in receipt of disability benefits and/or involved in litigation will show smaller effects than those not so involved;
(2) trials at high risk of bias will show larger effects compared to trials at low risk of bias;
(3) trials with longer follow-up will show smaller treatment effects;
(4) trials that enrolled patients formally diagnosed with PTSD according to validated criteria (eg, DSM-III, DSM IV, DSM IV-R, DSM-5, CAPS, or symptom severity measures) will show smaller effects than trials that do not require formal diagnosis;
(5) military and first-responder samples with PTSD will show smaller effects than civilian samples;
(6) patients with PTSD and co-morbid substance abuse will show smaller effects than patients with PTSD without co-morbid substance abuse;
(7) patients with PTSD and co-morbid traumatic brain injury will show smaller effects than patients with PTSD without co-morbid traumatic brain injury.

We have an additional a-priori to explore the small study effect we will run network meta-regression based on the SE.

### 2.10 Patient and public involvement

We have engaged an individual living with PTSD to help inform our research design, and when our review is completed we will ask them to assist in the interpretation and reporting of our findings. We plan to disseminate the results of our review to organizations supporting patients with PTSD, including Homewood Health and the Workers Compensation Board of Manitoba.

## 3 Discussion and knowledge translation

The results of our NMA will help clinicians and inform patients with PTSD about their therapeutic options, and facilitate informed health management decisions. The results of our review will be of interest to a broad audience including patients with PTSD, physicians, mental health clinicians, and third party payors – including insurers and compensation boards. Our review will identify key areas of future research and will provide a framework for conducting large systematic reviews involving indirect comparisons.

Our proposed review has several strengths. First, we will explore all currently available nonpharmacological and pharmacological treatment options for PTSD reported among eligible trials. Second, we will update the search to present date. Third, we will use the GRADE approach to evaluate the quality of evidence supporting treatment effects. Fourth, we will ensure interpretability by presenting risk differences and measures of relative effect for all pooled outcomes, and by presenting our findings with GRADE evidence profiles. Moreover, in order to explore the heterogeneity in our effect estimates, we have set 7 a-priori hypotheses and will conduct meta-regression and subgroup analyses consistent with best current practices.

The possible limitations of our review include the potential shortcomings of primary studies such as the presence of publication bias, high heterogeneity, and poor quality of reporting. Another likely limitation, unique to multiple treatment comparison meta-analyses, will be the nature of available treatment comparisons to build robust networks for our analyses.

## Author contributions

**Conceptualization:** Yasir Rehman, Behnam Sadeghirad, Jason Busse, Gordon Guyatt.

**Funding acquisition:** Jason Busse, Yasir Rehman, Gordon Guyatt.

**Methodology:** Yasir Rehman, Behnam Sadeghirad, Jason Busse, Gordon Guyatt.

**Supervision:** Yasir Rehman, Behnam Sadeghirad, Jason Busse.

**Writing – original draft:** Yasir Rehman, Behnam Sadeghirad, Jason Busse.

**Writing – review and editing:** Yasir Rehman, Behnam Sadeghirad, Jason Busse, Gordon Guyatt, Margaret McKinnon, Randi McCabe, Ruth Lanius, Donald Richardson.
